# Pathogenomes and virulence profiles of representative big six non-O157 serogroup Shiga toxin-producing *Escherichia coli*

**DOI:** 10.3389/fmicb.2024.1364026

**Published:** 2024-03-18

**Authors:** Anwar A. Kalalah, Sara S. K. Koenig, James L. Bono, Joseph M. Bosilevac, Mark Eppinger

**Affiliations:** ^1^Department of Molecular Microbiology and Immunology, University of Texas at San Antonio, San Antonio, TX, United States; ^2^South Texas Center for Emerging Infectious Diseases (STCEID), San Antonio, TX, United States; ^3^U.S. Department of Agriculture (USDA), Agricultural Research Service (ARS), U.S. Meat Animal Research Center, Clay Center, NE, United States

**Keywords:** Shiga toxin (Stx)-producing *Escherichia coli* (STEC), non-O157 big six serogroups, whole genome sequencing and typing (WGST), phylogenomics, virulence phenotyping

## Abstract

Shiga toxin (Stx)-producing *Escherichia coli* (STEC) of non-O157:H7 serotypes are responsible for global and widespread human food-borne disease. Among these serogroups, O26, O45, O103, O111, O121, and O145 account for the majority of clinical infections and are colloquially referred to as the “Big Six.” The “Big Six” strain panel we sequenced and analyzed in this study are reference type cultures comprised of six strains representing each of the non-O157 STEC serogroups curated and distributed by the American Type Culture Collection (ATCC) as a resource to the research community under panel number ATCC MP-9. The application of long- and short-read hybrid sequencing yielded closed chromosomes and a total of 14 plasmids of diverse functions. Through high-resolution comparative phylogenomics, we cataloged the shared and strain-specific virulence and resistance gene content and established the close relationship of serogroup O26 and O103 strains featuring flagellar H-type 11. Virulence phenotyping revealed statistically significant differences in the Stx-production capabilities that we found to be correlated to the strain’s individual *stx*-status. Among the carried Stx_1a_, Stx_2a_, and Stx_2d_ phages, the Stx_2a_ phage is by far the most responsive upon RecA-mediated phage mobilization, and in consequence, *stx_2a_ +* isolates produced the highest-level of toxin in this panel. The availability of high-quality closed genomes for this “Big Six” reference set, including carried plasmids, along with the recorded genomic virulence profiles and Stx-production phenotypes will provide a valuable foundation to further explore the plasticity in evolutionary trajectories in these emerging non-O157 STEC lineages, which are major culprits of human food-borne disease.

## 1 Introduction

Shiga toxin (Stx)-producing *Escherichia coli* (STEC) are distinguished from other *E. coli* pathovars (Kaper et al., 2004) by the production of a phage-borne cytotoxin (Smith et al., 2014; Krüger and Lucchesi, 2015; Zuppi et al., 2020) that is toxigenic toward renal endothelial (Obrig and Karpman, 2012) and intestinal epithelial cells (Schüller, 2011). *Escherichia coli* are historically classified by their variation in somatic O- and flagellar H-antigens (Orskov et al., 1977). Serotype O157:H7 is the dominant causative agent of STEC disease in the U.S. (Riley et al., 1983; Eppinger et al., 2011, 2013; Sanjar et al., 2014; Rusconi et al., 2016). However, the incidence of non-O157 infections that feature different somatic antigens has been steadily increasing in recent years (Gould et al., 2013; Vishram et al., 2021; Glassman et al., 2022; Tarr et al., 2023). Among these, emerging serogroups O26, O45, O103, O111, O121, and O145 account for the majority of clinical non-O157 STEC infections in the US and are colloquially referred to as the “Big Six” (Eklund et al., 2001; Johnson et al., 2006; Bettelheim, 2007; Hadler et al., 2011; Hegde et al., 2012; Gould et al., 2013; Vishram et al., 2021). Disease in humans can progress to life-threatening complications, such as hemolytic uremic syndrome (HUS) and ultimately renal failure (Karmali et al., 1983; Majowicz et al., 2014). The disease has been linked to the amount and subtype of toxin produced (Donohue-Rolfe et al., 2000; Russo et al., 2016). STEC can harbor one or multiple Stx-bacteriophages featuring different combinations of *stx*-suballeles (Krüger and Lucchesi, 2015; Rusconi et al., 2016) that can also form hybrid toxins (Skinner et al., 2014). The most potent cytopathic toxins, Stx_2a_ and Stx_2d_ (Fuller et al., 2011; Hauser et al., 2020; McNichol et al., 2021), are prevalent in the Big Six serogroups (Jinnerot et al., 2020), and a strain’s Stx-status is shaped by the dynamic Stx-phage acquisition, rather than by a common evolutionary history (Cowley et al., 2019; Nyong et al., 2020). Mobilization of Stx-prophages is triggered by diverse abiotic and biotic cues (Pacheco et al., 2012; Pacheco and Sperandio, 2012), and is required to produce toxin causing adverse toxigenic effects in murine STEC models (Nguyen and Sperandio, 2012; Tyler et al., 2013; Baumler and Sperandio, 2016; Balasubramanian et al., 2019; Rodríguez-Rubio et al., 2021). Triggering the RecA-dependent SOS-response with sublethal doses of mitomycin C (MMC) constitutes a major pathway of Stx_−_phage mobilization and is routinely used in public health laboratories to assess the pathogenic potential (Kimmitt et al., 2000). Besides Stx, another major virulence determinant is the locus of enterocyte effacement (LEE) packaged into a pathogenicity island, which encodes a type III secretion system (T3SS) along with its associated effectors, the outer membrane adhesin intimin (*eae*) and the translocated receptor (*tir*; Franzin and Sircili, 2015). The majority of the Big Six serogroups also carry serogroup-specific virulence plasmids along with an diverse array of additional plasmids (Caprioli et al., 2005; Ogura et al., 2009). In this study, we report the complete genomes and comprehensive analyses of the pathogenome composition along with Stx-production pathotypes of a Big Six reference strain panel representing each of the non-O157 STEC serogroups curated and distributed by the American Type Culture Collection (ATCC). The gathered pathogen information and recorded virulence traits provide a foundation to further elucidate the make-up and the evolutionary boundaries of these emerging non-O157 STEC.

## 2 Materials and methods

### 2.1 Bacterial strains analyzed in this study

Panel MP-9, a representative collection of clinical emerging non-O157 STEC strains, colloquially referred to as the “Big Six,” was obtained from the American Type Culture Collection (ATCC).^1^ Strains are of serotypes O26:H11 (BAA-2196), O45:H2 (BAA-2193), O103:H11 (BAA-2215), O111:H8 (BAA-2440), O121:H19 (BAA-2219), and O145:NM (BAA-2192). Isolates were sequenced to closure, and the culture’s virulence was profiled in this study. Accessions for genomic reads, assembled annotated chromosomes and plasmids along with strain-associated metadata are provided in Table 1 and Supplementary Table S1.

**Table 1:** Molecules and accessions.

ATCC Strain	Serotype	Chromosome accessions	Plasmids	Plasmid accessions
BAA-2192	O145:H-	CP101310	pO145	CP101311
BAA-2440	O111:H8	CP101307	pCol156-O111-1	CP101308
			pCol-O111-2	CP101309
BAA-2219	O121:H19	CP101305	pO121	CP101306
BAA-2193	O45:H2	CP101302	pO45-1	CP101303
			pO45-2	CP101304
BAA-2215	O103:H11	CP101298	pO103-1	CP101300
			pO103-2	CP101301
			pCol-O103-3	CP101299
BAA-2196	O26:H11	CP101292	pO26-1	CP101295
			pO26-2	CP101296
			pCol-O26-3	CP101294
			pO26-4	CP101297
			pCol156-O26-5	CP101293

### 2.2 Genome sequencing, assembly, and annotation

Strains were cultured overnight at 37°C with shaking at 220 rpm in lysogeny broth (LB; Thermo Fisher Scientific, Asheville, NC, United States). To maximize total genomic DNA (gDNA) yields, bacterial overnight cultures were diluted to OD_600_ of 0.03 in fresh LB medium and grown at 37°C with shaking at 220 rpm to mid-log phase (OD_600_ ~ 0.5). Total gDNA was extracted using the Qiagen Genomic-tip 100/G Kit (Qiagen, Inc., Valencia, CA, United States) according to the manufacturer’s instructions. Genomic DNA was subjected to both long-read (Oxford Nanopore, Oxford, United Kingdom) and short-read (Illumina, Inc., San Diego, CA, United States) sequencing. For long-read Nanopore sequencing, gDNA was diluted to a concentration of 1.5 μg in 46 μL of nuclease-free water. The library was prepared using the Ligation Sequencing Kit (SQK-LSK109) with the Native Barcoding Expansion 1–12 (EXP-NBD104) according to the manufacturer’s instructions and sequenced on a MinION with the R10.3 SpotON Flow Cell (FLO-MIN111). Paired-end short-read libraries were prepared with the Illumina Nextera XT DNA Library Preparation Kit and sequenced on the MiSeq platform using the MiSeq reagent Kit (v3) with 600-cycles. Sequence reads in the fastq format were imported into Galaxy v.22.05 (Community, 2022). Default parameters were used for all software unless specified otherwise. Quality control of fastq files was assessed using FastQC (v.0.74 + Galaxy0).^2^ Nanopore and Illumina reads were used for hybrid assembly using Unicycler assembler (v.0.5.0 + Galaxy1; Wick et al., 2017). The chromosomal *dnaA* and plasmid *repA* genes, if applicable, were designated as the zero point of the closed molecules prior to annotation using the NCBI Prokaryotic Genome Annotation Pipeline (PGAP; Tatusova et al., 2016).

### 2.3 Pathogenome make-up and visualization

Chromosomes and plasmids were comprehensively analyzed and visualized in Blast Ring Image Generator BRIG (v.0.95; Alikhan et al., 2011) and MAUVE (v.2.4.1; Darling et al., 2008, 2010). Serotypes in the assembled genomes were confirmed *in silico* using the EcOH database (Ingle et al., 2016) in ABRicate (Galaxy v.1.0.1)^3^ with options—minid 80—mincov 80 (Community, 2022). Average nucleotide identities (ANI) using the *E. coli* strain BAA-2196 (O26:H11) chromosome as designated reference were calculated with FastANI (Galaxy v.1.3), based on MinHash mapping (Jain et al., 2018). Chromosomal repeats were identified with FindRepeats (v.1.8.2 + Galaxy1; Kurtz et al., 2004; Petkau et al., 2017). Virulence and antibiotic resistance genes (ARGs) were identified using VFDB (Liu et al., 2022) and ResFinder^4^ (Florensa et al., 2022), respectively. Boundaries and locations of intact, partial, or remnant prophages were identified using PHASTER (Zhou et al., 2011; Arndt et al., 2016) and MAUVE (v.2.4.1; Darling et al., 2008, 2010), followed by manual curation with BLASTn/p against the non-redundant NCBI databases (Camacho et al., 2009). Toxin subtypes of the carried Stx-bacteriophages were recorded *in silico* as described elsewhere by blastn of the carried toxins against an *stx* suballele database (Scheutz et al., 2012; Ashton et al., 2015; Carrillo et al., 2016). The EHEC phage replication unit (*eru*) subtype was assigned as described in Llarena et al. (2021) and Fagerlund et al. (2022) and Stx-prophages genomes were visualized in Easyfig (v.2.2.2; Sullivan et al., 2011). Mechanistics of phage insertion can create direct repeats (DR) and insertion sites were investigated for direct repeats (DR) and attachments sites (*att*) using NUCmer (v.4.0.0rc1 + Galaxy2) and BLASTn (Camacho et al., 2009). Lytic phage loci in ΦStx- and non-ΦStx-prophages were identified with Prophage Hunter (Song et al., 2019). Insertion sequence (IS) elements were identified and curated using ISEScan (v.1.7.2.3 + Galaxy0; Xie and Tang, 2017). Integrons were surveyed with Integron Finder (v.2.0.2 + Galaxy1; Néron et al., 2022). Genomic islands (GI) were detected with IslandViewer4 (Bertelli et al., 2017, 2018; Bertelli and Brinkman, 2018). Plasmid incompatibility groups were identified and analyzed with MOB-Typer (v.3.0.3 + Galaxy0; Robertson and Nash, 2018).

### 2.4 Shiga toxin and intimin subtyping

For toxin subtyping, the *stx* genes were aligned to a multifasta file comprised of all currently published *stx*-suballele nucleotide sequences (Scheutz et al., 2012; Carrillo et al., 2016; Bai et al., 2018; Yang et al., 2020) with BLASTn (Camacho et al., 2009). Heatmaps of cataloged genes were generated with iTol (v.6.8.1; Letunic and Bork, 2021). LEE islands were identified starting from the LEE1 operon gene *espG* to the *espF* gene in LEE4, and their comparative analysis was conducted and visualized using GeneSpy (Garcia et al., 2019). We determined the subtypes by aligning the intimin genes to the 27 currently published subtype sequences of *eae* in GenBank (α1-2, β1-3, γ, δ, ε1-4, ζ1 and 3, η1-2, θ1-2, ι1-2, κ, μ, ν, ξ, ο, π, ρ, σ; Supplementary Table S2) using BLASTn (Camacho et al., 2009).

### 2.5 MLST schemas and phylogenetic analyses

The assembled ATCC MP-9 genomes along with *E. coli* strains EC4115 (O157:H7; Eppinger et al., 2011) and K-12 substrain MG1655 (Blattner et al., 1997) were imported into SeqSphere+ (v.8.3; Ridom GmbH, Münster, Germany) for gene-by-gene alignment, allele calling, and comparison (Jünemann et al., 2013). MLST typing was performed using targeted and whole genome schemas developed for *E. coli* (Foley et al., 2009; Zhou et al., 2020). We determined the Sequence Type (ST) by applying the 7-gene ST Achtman schema (Zhou et al., 2020). Allele sequences for the 7 genes (*adk*, *fumC*, *gyrB*, *icd*, *mdh*, *purA*, and *recA*) were accessed on the EnteroBase website^5^ and imported into Ridom SeqSphere+. A core genome (cg) MLST schema was developed using the closed chromosome of K-12 substrain MG1655 (GenBank accession U00096; Riley et al., 2006) as seed as previously described (Díaz et al., 2021). Core and accessory MLST targets were identified according to the inclusion/exclusion criteria of the SeqSphere+ Target Definer. The allele information from the targeted seven-gene schema and the defined core genome gene of the panel strains were used to establish phylogenetic hypotheses using the minimum-spanning method (Kruskal, 1956; Francisco et al., 2009) with default settings in Ridom SeqSphere+ (v.8.3).

### 2.6 Growth of cultures in LB and under phage mobilizing condition in LB + MMC

Strains were cultured overnight (o/n) at 37°C with shaking at 220 rpm in LB. Overnight LB cultures were diluted to an OD_600_ of 0.03 in fresh LB media, grown to early-log phase (OD_600_~0.3) at 37°C, and then subdivided into two subcultures, LB and LB + MMC. Triggering the RecA-dependent SOS-response with MMC constitutes a major pathway of Stx_−_phage mobilization (Kimmitt et al., 2000). Subculture LB + MMC was supplemented with MMC (Sigma-Aldrich, Saint Louis, MO, United States) at a final concentration of 0.5 μg/mL to mobilize the carried prophages, while subculture LB was used to evaluate spontaneous prophage mobilization. To confirm phage mobilization in MMC-treated cultures, growth curves were recorded in a 96-well plate (Corning 3,370, Corning Inc., Corning, NY, United States) on a BioTek Synergy H1 plate reader (BioTek Instruments, Inc., Winooski, VT, United States) recording OD_600_ values for 6 h at 10 min intervals. All experiments were executed in two biological replicates.

### 2.7 Virulence phenotypes

#### 2.7.1 PCR experiments

Primers and PCR-conditions are provided in Supplementary Table S3. LB and LB + MMC subcultures were grown for 6 h at 37°C with shaking at 220 rpm and then centrifuged at 5,000 g for 10 min: (1) Cell pellets were used to determine *stx*-transcripts levels, while (2) the supernatants were used to enumerate ΦStx-phage copies, targeting the phage-borne *stx* loci as follows: (1) Expression of *stx* genes RNA was purified using the PureLink RNA Mini kit (Invitrogen, Waltham, MA, United States), and RNA quantity and quality were measured with the NanoDrop ND-1000 Spectrophotometer (Thermo Fisher Scientific, Waltham, MA, United States). Total RNA was treated with amplification grade DNase I (Invitrogen, Waltham, MA, United States), and reverse transcribed using the RevertAid H Minus First Strand cDNA Synthesis Kit (Thermo Fisher Scientific, Waltham, MA, United States). The *stx*-RT-qPCR was performed on the StepOne Real-Time PCR System (Applied Biosystems, Foster City, CA, United States) using the GoTaq qPCR Master Mix (Promega, Madison, WI, United States). (2) Enumeration of ΦStx_1_- and ΦStx_2_-phage copies Supernatants were filtered through low-protein-binding 0.22-μm-pore-size membrane filters (Millex-GP; Merck Millipore Ltd., Burlington, MA, United States), followed by DNase I (Invitrogen, Waltham, MA, United States) treatment for 15 min to remove bacterial gDNA. Lysate phage DNA was isolated using the QIAamp DNA Mini Kit (Qiagen Inc., Valencia, CA, United States), and eluted with 50 μL nuclease-free water. Phage numbers were determined by *stx*-qPCR on the StepOne Real-Time PCR System (Applied Biosystems, Foster City, CA, United States) using the GoTaq qPCR Master Mix (Promega, Madison, WI, United States). Standard curves for the *stx* transcripts and ΦStx-phage copy numbers were calculated using gBlocks (Integrated DNA Technologies (IDT), Coralville, Iowa, United States) in the RT-qPCR and qPCR experiments, respectively.

#### 2.7.2 Stx-production pathotypes

The Stx-production phenotypes of the cultures were determined by recording the Stx titers through Enzyme-Linked ImmunoSorbent Assay (ELISA) under both spontaneous and MMC-induced conditions. Overnight (o/n) cultures were diluted to an OD_600_ of 0.03 and grown to early-log phase (OD_600_~0.3) in replenished LB media at 37°C. At this stage, cultures were split and incubated at 37°C for 6 h under non-induced and induced (0.5 μg/mL MMC) conditions. Toxin production was measured after harvesting 5 mL of each culture for parallel processing. To lyse bacterial cells and release produced Stx, cultures were treated with polymyxin B (Sigma-Aldrich, Saint Louis, MO, United States; 6 mg/mL 37°C, 10 min). Supernatants were collected after centrifugation (3,500 rpm, 10 min), filtered through 0.22 μm low protein-binding membrane filters (Millex-GP; Merck Millipore Ltd., Burlington, MA, United States) and diluted to measurable concentrations. Stx-production was measured using the Premier EHEC kit (Meridian Bioscience, Cincinnati, OH, United States) following the manufacturer’s instructions. Titers were calculated using a standard curve generated from serial dilutions of purified Stx_2a_ (BEI Resources, NR-4478). Statistical significance was determined using Prism (v.9.5.0; GraphPad Software, San Diego, CA, United States). A two-way ANOVA with Sidak’s multiple comparisons test was used to compare non-induced to MMC-induced conditions for each strain. Strain-to-strain comparisons were performed with a one-way ANOVA with Tukey’s multiple comparisons test assessing each condition.

## 3 Results

### 3.1 Pathogenome composition of MP-9 panel strains

**Figure 1:**
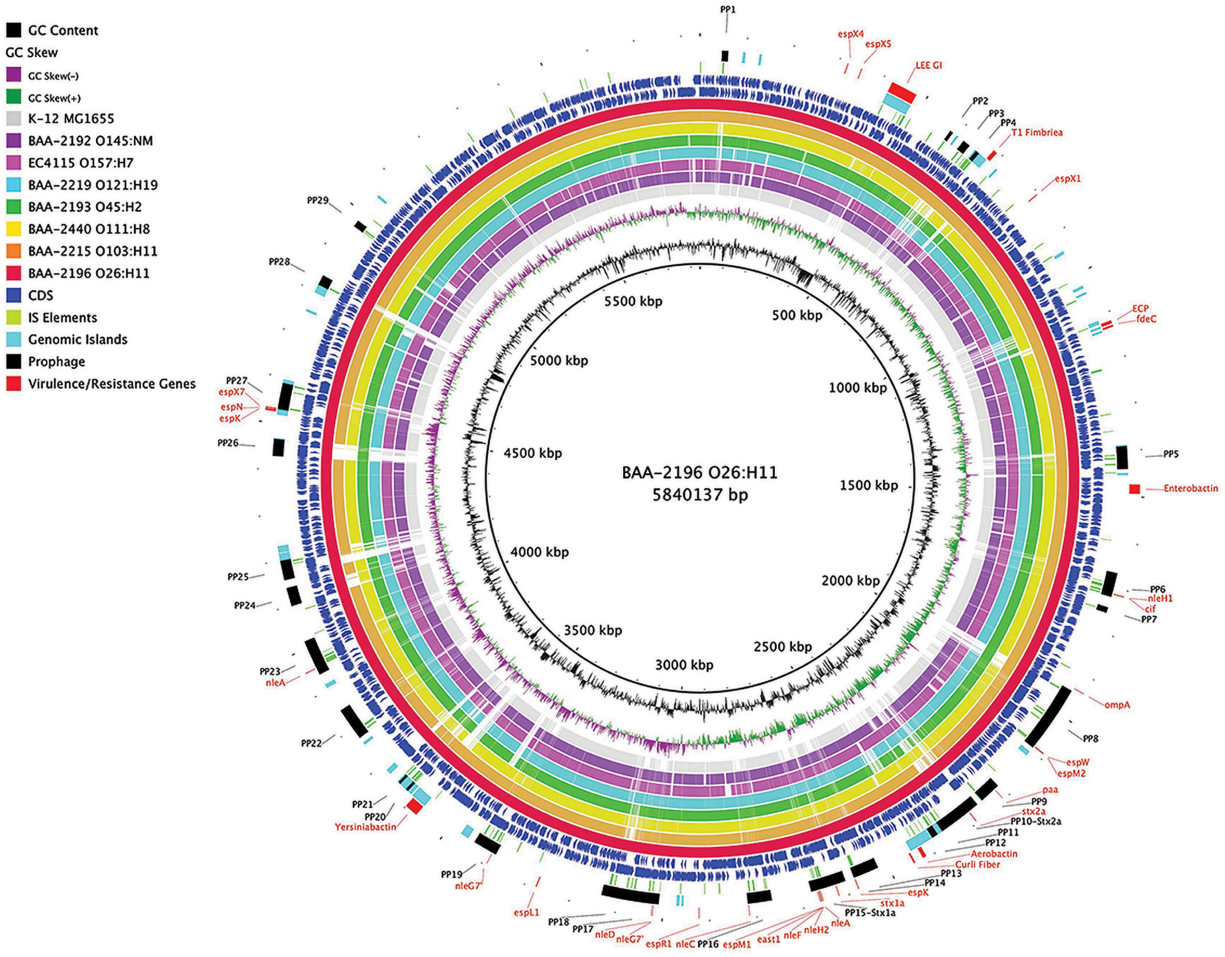
Comparison of ATCC MP-9 panel genomes BRIG comparison of six sequenced strains, along with *Escherichia coli* strains O157:H7 EC4115 and strain K-12 substrain MG1665, referenced to the 5,840,137 bp chromosome of BAA-2196 (O26:H11). CDS are presented on the +/−strands as blue arrows and functional annotations for virulence genes and other loci of interest are highlighted as shown in the legend. Query genomes are color-coded, and the order plotted in the circle reflects the inferred phylogenomic relationships.

**Figure 2:**
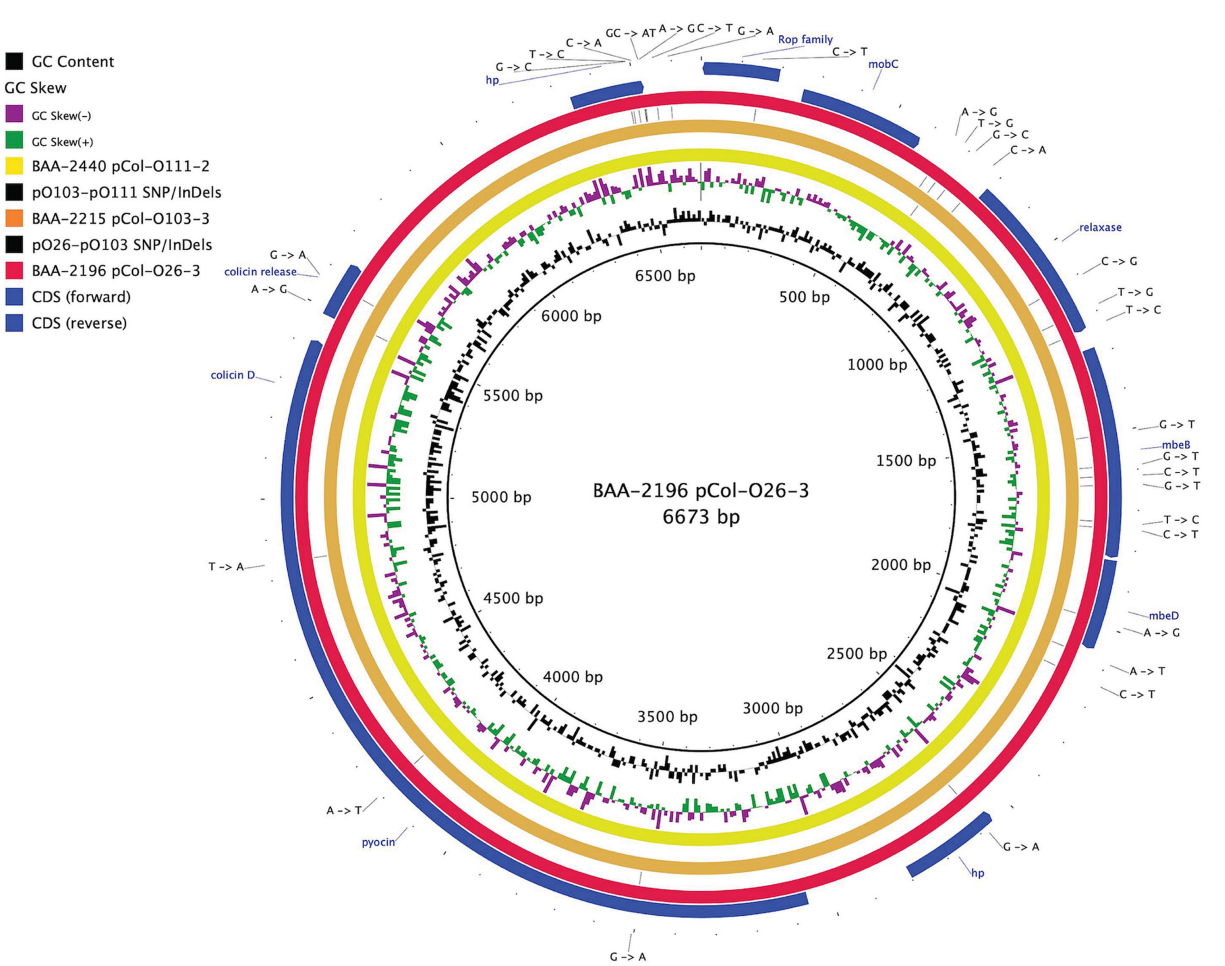
Comparison of a shared colicin plasmid BRIG comparison of a shared colicinogenic plasmid l present in serotypes O26:H11, O103:H11, and O111:H8 and referenced to the 6,673 bp plasmids of BAA-2196 pCol-O26-3. The plasmids are differentiated by a total of 30 SNPs and InDels. CDS are presented on the +/− strands as blue arrows. Query plasmids are plotted according to the strain’s inferred phylogenomic relationships.

In this study, we sequenced and comprehensively analyzed the pathogenomes and virulence traits of six non-O157 STEC strains. Strain panel MP-9 was obtained from ATCC, which is comprised of six strains representing each of the non-O157 STEC serogroups, colloquially referred to as the “Big Six” (Eklund et al., 2001; Johnson et al., 2006; Bettelheim, 2007; Hadler et al., 2011; Hegde et al., 2012; Gould et al., 2013; Vishram et al., 2021; Supplementary Table S1). STEC genomes house an extensive and partly repetitive phage complement that hampers assembly into closed genomes (Goldstein et al., 2019; Jaudou et al., 2022). In response, we applied a long- and short read sequencing hybrid strategy (Nyong et al., 2020; Allué-Guardia et al., 2022) that allowed us to provide the high-quality closed genomes, including carried plasmids (Figures 1, 2; Supplementary Figures S1, S2). The chromosomes have an average nucleotide identity of 98.8%, with a range from 97.6% to 99.8%, indicative of the substantial conserved chromosomal backbone of *E. coli* (Rasko et al., 2008; Jain et al., 2018). The chromosome size in this panel ranges from 5,288,508 to 5,840,137 bp with an average GC-content of 50.65%. When compared to non-pathogenic *E. coli* strain K-12 substrain MG1665, these STEC strains carry at least 648,833 bp of additional genetic information. Genome statistics along with strain-associated metadata are provided in Supplementary Table S1. In Figure 1, we compared the chromosomes using strain BAA-2196 (O26:H11) as the designated reference. In comparison to *E. coli* strain K-12, the Big Six strains acquired multiple mobile genome elements (MGE), including the hallmark ΦStx-prophages, which are major contributors of STEC genome evolution and diversification (Lawrence and Ochman, 1998; Rasko et al., 2008; Robins-Browne et al., 2016). Individual comparisons referenced to each of the strains can be found in Supplementary Figure S1. The mobilome on the chromosomes consisting of prophages, genomic islands, and IS elements contributes 22.4% to 28.7% of sequence information, in line with the assessment in other STEC (Perna et al., 2001; Delannoy et al., 2017; Supplementary Table S4). Neither chromosomal nor plasmid-borne integrons were detected. The prophages account for 13.9 to 21.2% of the chromosome, followed by genomic islands (5.4 to 6.9%), and IS elements (0.8 to 2.5%). If plasmid-carried IS elements are considered, the percentage of IS elements increases by 1.2 to 2.7%. The IS elements in this panel showed variations in both prevalence and numbers (Supplementary Table S4). ISEScan detected 726 IS elements and categorized them into 16 known families and 40 clusters, indicative of the plasticity present in these non-O157 STEC (Supplementary Figure S3). Eight of the 40 clusters were present in the six isolates, though their respective numbers between the strains vary considerably. We further note that BAA-2196 (O26:H11) and BAA-2215 (O103:H11) strains feature similar copy numbers in shared IS clusters distinct from the remainder of strains indicative of their close relationship (Iguchi et al., 2012; Ju et al., 2012; Supplementary Figure S3; Supplementary Table S4). Thirteen elements of the IS*3-168* cluster were found in each of BAA-2196 (O26:H11) and BAA-2215 (O103:H11), compared to an average of 55 copies in other strains. Inversely, the IS*66-46* cluster was found to have 48 and 36 copies in BAA-2196 and BAA-2215, respectively, while other strains carry an average of eight copies. Further, eight clusters are strain-specific, and 24 clusters are present in a subset of strains. This may suggest different dynamics in the propagation of these elements.

### 3.2 Phylogenomic relatedness of ATCC MP-9 strains

**Figure 3:**
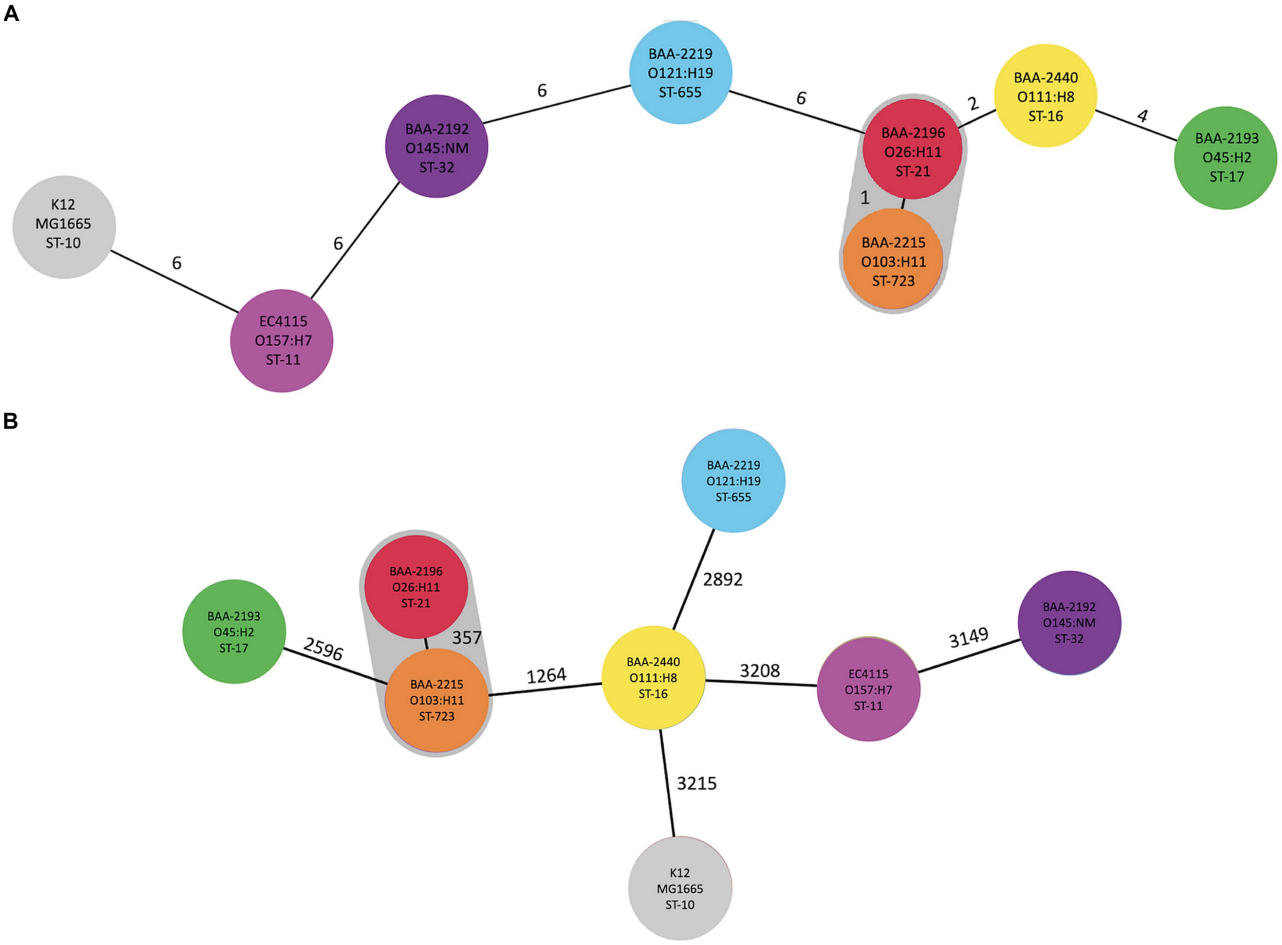
Phylogenomic position of ATCC-MP9 strains The relatedness of panel strains, including O157:H7 strain EC4115, was determined using MLST in Ridom SeqSphere+: **(A)** targeted seven-gene MLST accessed in EnteroBase. Numbers on connecting branches indicate the number of genes with differing allele status, and **(B)** cgMLST-based phylogeny using the closed chromosome of *E. coli* strain K12 subst. MG1655 as seed. The shared gene inventory was determined at 4,304 genes, according to the inclusion/exclusion criteria of the SeqSphere+ Target Definer and is comprised of 3,148 core and 908 accessory loci. Colors denote ST-classifications established in **(A)**.

The mobilome is comprised of prophages, genomic islands, IS elements, and plasmids, which evolve at different rates and can be acquired and secondarily lost and thus are often not indicative of evolutionary relationships. To investigate the phylogenomic boundaries of the individual strains, we established a phylogenomic framework inferred from targeted MLST and core genome MLST (cgMLST; Figure 3). As expected for this heterogenous set of serotypes, the strains belong to distinct STs with a total of 14,340 allelic changes and 926 InDels (Figure 3; Supplementary Table S5). Their shared inventory was computed at 4,304 genes comprised of 3,148 core and 908 accessory loci, indicative of the extended conserved *E. coli* backbone (Abu-Ali et al., 2009; Lim et al., 2010; Eppinger et al., 2011; Yin et al., 2015). High-resolution core genome MLST typing revealed a close phylogenetic relationship of serogroup O26:H11 and O103:H11 strains, as previously suggested by MLST- and genome-wide single nucleotide polymorphisms (SNPs)-based analyses for these serogroups carrying flagellar antigens H2 and H11 (Iguchi et al., 2012; Ju et al., 2012). ST-21 (BAA-2196 O26:H11) and ST-723 (BAA2215 O103:H11) are only separated in their *fumC* allele and 357 allelic changes in the cgMLST analysis (Figure 3). This intimate relationship is reflected in the isolates’ shared chromosomal and mobilome inventories, such as virulence genes, prophages, and LEE island organization, as discussed below.

### 3.3 Comprehensive analyses of plasmid content and function

**Figure 4:**
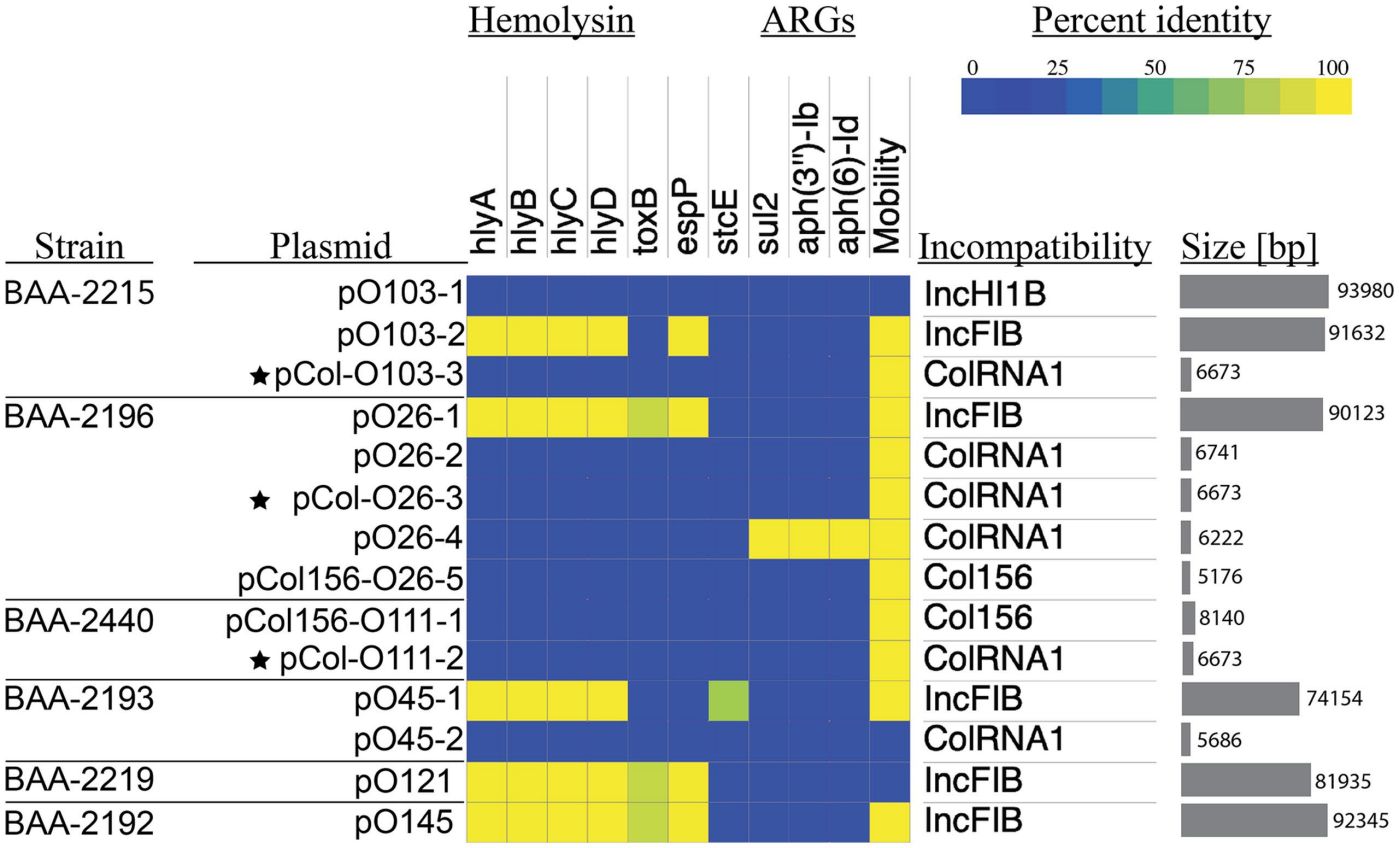
Prevalence and distribution of plasmid-borne virulence determinants Percentage identities of virulence and antimicrobial resistance genes identified in VFDB and ResFinder are visualized in a heatmap. The Plasmid incompatibility group and predicted mobility were determined with MobTyper. The shared colicinogenic plasmid pCol present in the serotype O26:H11, O103:H11, and O111:H8 strains is indicated with a star.

The hybrid-sequencing strategy further identified 14 functionally and phylogenetically diverse plasmids that range in size from 5,176 to 93,980 bp and belong to four incompatibility groups (Supplementary Figure S2; Supplementary Table S1). STEC often carry plasmids that contribute diverse virulence determinants (Kaper et al., 2004; Pilla and Tang, 2018). Virulence plasmids coding for hemolysin (*hlyCABD*), adhesin (*toxB*), and serine protease (*espP*) were found in all strains, except in strain BAA-2440 O111:H8 (Tatsuno et al., 2001; Kaper et al., 2004; Caprioli et al., 2005; Tozzoli et al., 2005; Johnson and Nolan, 2009; Figure 4). Colicins are synthesized to gain an advantage in the shared niche and are toxic to other bacterial strains (Cascales et al., 2007). Three strains, BAA-2440, BAA-2196, and BAA-2215, contained colicinogenic plasmids. Strain BAA-2440 O111:H8 codes for colicins E3 and D on plasmids pCol156-O111-1 and pCol-O111-2, respectively. The latter is phylogenetically related to plasmids pCol-O26-3 and pCol-O111-2 exhibiting a highly conserved plasmid backbone differentiated from each other by 30 SNPs and InDels (Figure 2). A Blastn query against the NCBI non-redundant database found related plasmids in Big Six serogroups O26, O103, and O111, and STEC serogroups O104, O157, and O165, among others, as shown in Supplementary Table S4 (Ogura et al., 2009; Yan et al., 2015; Sekizuka et al., 2019; Amadio et al., 2021). Strain BAA-2196 O26:H11 carries plasmid pO26-4, which is a multidrug-resistant plasmid encoding three ARGs (*sul2*, *aph(6)-Ib*, and *aph(3″)-Ib*) conferring resistance to sulfonamide and aminoglycosides (Hammerum et al., 2006; Bean et al., 2009; Messele et al., 2022; Supplementary Figure S2A; Supplementary Table S4). This broad host range plasmid shares high nucleotide similarity (>99%) and coverage (>99%) to plasmids found in *E. coli*, *Shigella* sp., *Citrobacter freundii* and *Klebsiella pneumoniae* (Iguchi et al., 2009; Ye et al., 2010; Kyle et al., 2012; Liu et al., 2015; AbuOun et al., 2021; Supplementary Table S4).

### 3.4 Comprehensive analyses of virulence determinants and Stx-status

**Figure 5:**
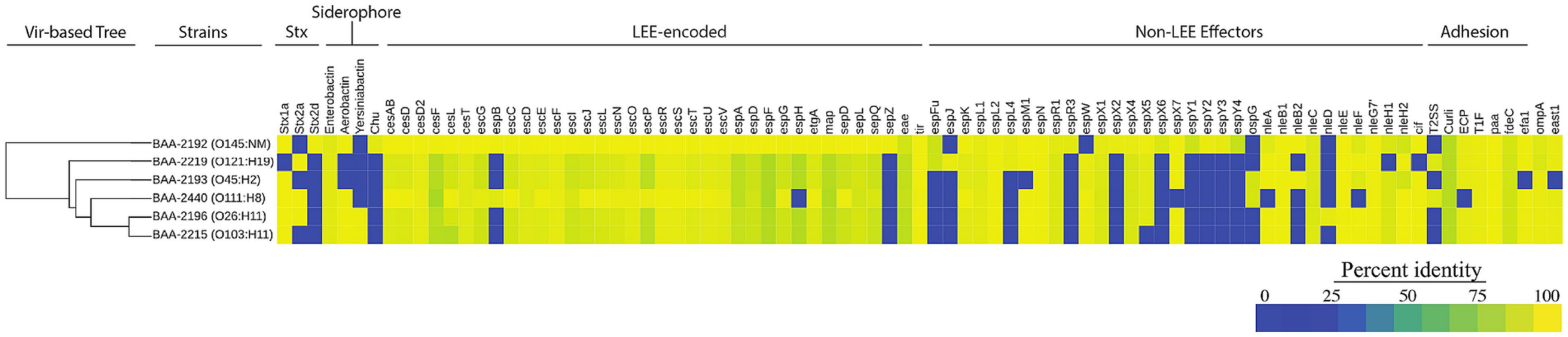
Prevalence and distribution of chromosomal virulence determinants Percentage identities for each virulence gene identified in VFDB are visualized in a heatmap. The panel strains encode a total of 149 distinct virulence genes of which 113 are shared. Among these are toxin suballeles *stx_1a_*, *stx_2a_*, and *stx_2d_*, the LEE genomic island, and siderophores, among others. The strain order reflects the inferred phylogenomic relationships. The hierarchical clustering of virulence genes based on their pair-wise distance is shown on the left.

The prevalence of the identified chromosomal and phage- and plasmid-borne virulence genes revealed a considerable plasticity in the individual virulence complement. We surveyed chromosomes for virulence and resistance loci and analyzed their prevalence and distribution (Figure 5; Supplementary Table S6). In total, we identified 149 chromosomal virulence genes of which 113 are shared by all strains (Figure 5). The latter includes the phage-borne *stx*, along with genes that make up the LEE including its effectors (Moon et al., 1983; Mellies et al., 1999; Jores et al., 2004; Kaper et al., 2004; Sadiq et al., 2014; Franzin and Sircili, 2015). The strains feature four distinct siderophore types that facilitate iron acquisition in the iron limiting condition of mammalian hosts (Ratledge and Dover, 2000; Skaar, 2010; Caza and Kronstad, 2013; Sheldon et al., 2016). All strains possess enterobactin (*ent*), widely distributed in *E. coli* (Cox et al., 1970; Garcia et al., 2011; Mey et al., 2021). Yersiniabactin (*ybt*) and hydroxamate aerobactin (*iuc*) are present in the phylogenetically related strains BAA-2196 (O26:H11) and BAA-2215 (O103:H11; Iguchi et al., 2012; Ju et al., 2012; Figure 3). Hydroxamate aerobactin is also found in strains BAA-2440 (O111:H8) and BAA-2192 (O145:NM), and the heme utilization operon (*chu*) in BAA-2192 (O145:NM; Figure 5). We note here that siderophores such as *ybt* and *chu* have been proposed biomarkers for serotypes O26, O157, and O145 (Pasquali et al., 2018; Jarocki et al., 2019; Carbonari et al., 2022). Antimicrobial-resistant STEC, though uncommon, have been isolated from humans, animals, and food (Eppinger et al., 2011; Amézquita-López et al., 2016; Mukherjee et al., 2017; Greig et al., 2023; Lee et al., 2023). The ATCC MP-9 strains do not carry any chromosomal antimicrobial resistance loci other than the efflux pump gene *mdf(A)* (Edgar and Bibi, 1997), found in most *E. coli* (Ahmed et al., 2020; Moser et al., 2021; Zhou et al., 2022; Awosile et al., 2023; Lee et al., 2023).

### 3.5 Comprehensive analysis of Stx-phages

**Figure 6:**
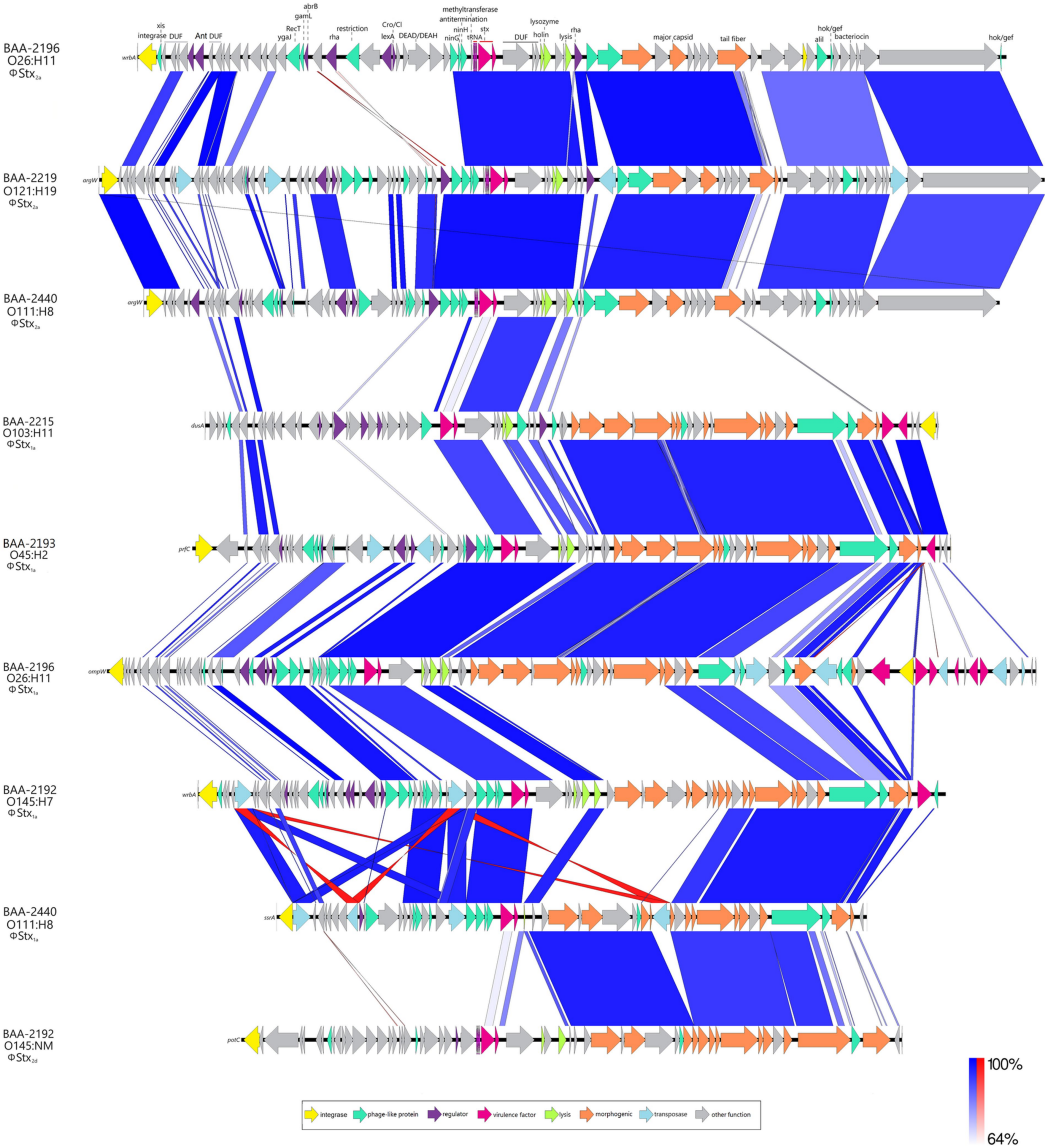
Comparison of Stx-prophages BLASTn-based comparison of architectures and content of carried ΦStx_2a_-, ΦStx_1a_-, and ΦStx_2d_-prophages. For uniform annotation, we inferred the annotation from the curated ΦStx_2a_-prophage genome of strain BAA-2196 (O26:H11).

Carriage of ΦStx-prophages is a virulence hallmark of STEC; and genomes can contain multiple ΦStx-prophages in diverse *stx*-suballele combinations (Huang et al., 1987; Eppinger et al., 2011; Krüger and Lucchesi, 2015; Rusconi et al., 2016; Allué-Guardia et al., 2022). Stx is a key virulence factor responsible for the severe symptoms associated with STEC infections such as HUS (Karmali et al., 1983). The panel strains carry either one or two ΦStx-prophages featuring suballeles *stx_1a_*, *stx_2a_*, and *stx_2d_* (Figures 5, 6). Two suballeles have been associated with elevated cytotoxicity, *stx_2a_* (Fuller et al., 2011; Hauser et al., 2020; Pinto et al., 2021) and *stx_2d_* (McNichol et al., 2021). Suballele *stx_2a_* was found alone (BAA-2219 O121:H19) or in combination with *stx_1a_* (BAA-2196 O26:H11, BAA-2440 O111:H8). Two strains carry *stx_1a_* only (BAA-2215 O103:H11, BAA-2193 O45:H2), or in combination with *stx_2d_* (BAA-2192 O145:NM). As evident in the comparison of the individual subtypes (Figure 6), the prophages show a high degree of genomic plasticity, in particular upstream of the toxin locus, important for regulation and replication (Unkmeir and Schmidt, 2000; Yin et al., 2015). Variability in these regions has been linked to strain-level differences in Stx-production (Herold et al., 2004; Smith et al., 2014; Ogura et al., 2015; Yin et al., 2015; Llarena et al., 2021; Rodríguez-Rubio et al., 2021; Fagerlund et al., 2022; Zhang et al., 2022; Yano et al., 2023). In total, seven chromosomal sites are occupied (Supplementary Table S4), some of which are known ΦStx-phage targets (Serra-Moreno et al., 2007; Eppinger et al., 2011; Bonanno et al., 2015; Rusconi et al., 2016; Allué-Guardia et al., 2022). The ΦStx-phage integrases have undergone evolution that allows them to target distinct insertion sites. Stx-phages tend to primarily integrate at a specific site; however, the integrase demonstrates the capacity to detect alternate insertion sites for integration if the preferred site is already occupied or absent (Groth and Calos, 2004; Serra-Moreno et al., 2007; Casjens and Hendrix, 2015; Henderson et al., 2021). ΦStx_2a_ phages are inserted into either arginine tRNA *argW* or NAD(P) H dehydrogenase *wrbA*, and the ΦStx_1a_ phage, in analogy to some ΦStx_2a_ in *wrbA*, or alternatively in peptide chain release factor *prfC*, outer membrane protein *ompW*, the tRNA-dihydrouridine synthase *dusA*, or tmRNA *ssrA*, while the ΦStx_2d_ phage is disrupting the spermidine uptake gene *potC* (Figure 6). As evident in the occupation status of *wrbA* by either ΦStx_1_ or ΦStx_2_, there is no association between toxin suballele and insertion sites in line with previous observation (Groth and Calos, 2004; Serra-Moreno et al., 2007; Steyert et al., 2012; Henderson et al., 2021).

### 3.6 Comprehensive analyses of the locus of enterocyte effacement

**Figure 7:**
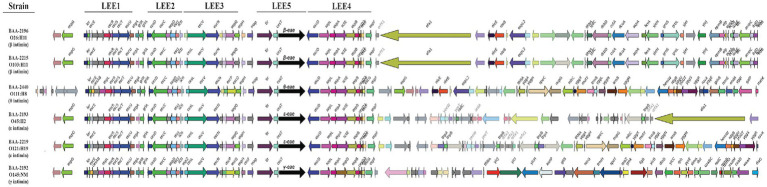
Comparison of LEE islands The complete LEE islands were extracted and compared in GeneSpy. Genes are colored according to their nucleotide homologies. The organization of the LEE1 to 5 operons is indicated above. The order of strains reflects their inferred phylogenomic position and is mirrored in the LEE island organization.

Carriage of the LEE pathogenicity island is responsible for the development of the characteristic attaching and effacing (A/E) lesions (Jerse et al., 1990; McDaniel et al., 1995; Sperandio et al., 1998; Schmidt, 2010; Stevens and Frankel, 2014; Franzin and Sircili, 2015). It is organized into polycistronic operons, LEE1 to 5, encoding T3SS components and regulators, chaperones, and effectors (Jerse et al., 1990; Mellies et al., 1999; Kirsch et al., 2004; Schmidt, 2010). Among the LEE-encoded proteins is intimin (Eae), an outer membrane adhesin that mediates the intimate bacterial attachment to the host’s intestinal cells (Supplementary Table S2). We detected *eae* subtypes β, ε, γ, and θ, and further located the respective boundaries of the islands (Figure 7). The LEE operon organization is conserved with minor rearrangements in BAA-2440 O111:H8 at *espG*/*rorf1* as previously described in the O111:H- serotype (Ogura et al., 2009). As evident in Figure 7, the LEEs of β-*eae* + strains BAA-2196 O26:H11 and BAA-2215 O103:H11 exhibit syntenic organization and inventory, again suggesting a close relationship as established by our cgMLST analyses (Figure 3).

### 3.7 Comparison of Stx-virulence pathotypes

**Figure 8:**
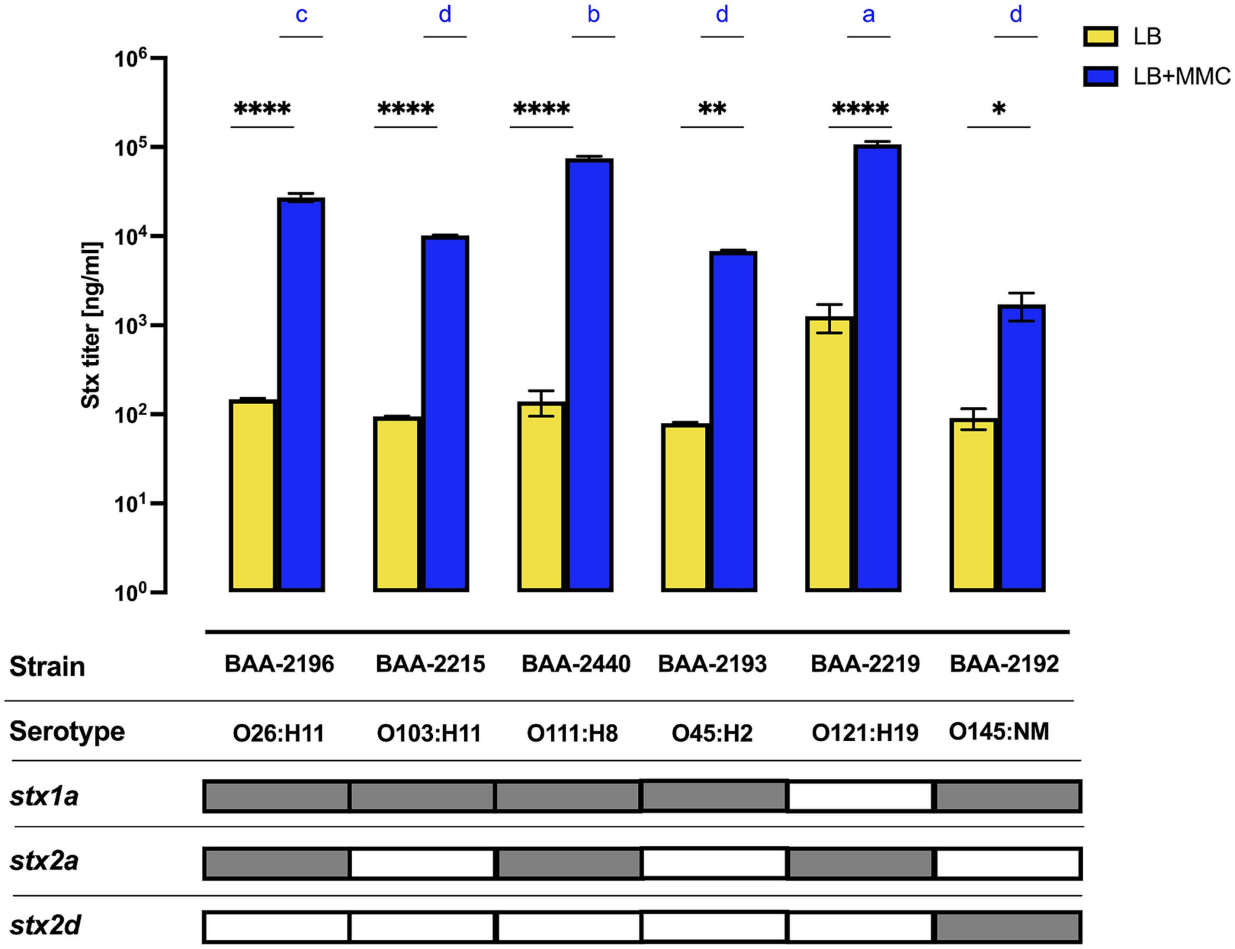
Variability in Stx-production The concentration of Stx_1_ and Stx_2_ produced under non-induced and MMC-induced phage mobilizing conditions was quantified by ELISA. Differences between the non-induced and MMC-induced phage mobilizing conditions for each strain were assessed using a two-way ANOVA, followed by Sidak’s multiple comparisons. Statistical significance is denoted as *p < 0.05; **p < 0.005; ***p < 0.0005; and ****p < 0.00005. For strain–strain comparison under MMC-induced conditions, differences in toxin concentration are indicated by letters (a–d), with “a” denoting the highest concentration group, in a descending order determined by a one-way ANOVA with Tukey’s multiple comparisons test.

The actual disease outcome cannot be predicted from *in silico* virulence profiling, considering the complex interactions between infective agent, the host microbiota (Pruimboom-Brees et al., 2000; Gamage et al., 2006; Nguyen and Sperandio, 2012), and the infected patient (Wong et al., 2000; Dundas et al., 2001; Gould et al., 2009; Foster, 2013). Induction efficiency of the Stx-phages is positively correlated to Stx-production (Muniesa et al., 2004; Loś et al., 2009; Del Cogliano et al., 2018) and thus mobilization of Stx-phages is used as a means to assess the conferred pathogenic potential (Karch et al., 1999; Eppinger et al., 2022; Miyata et al., 2023). For the panel cultures, we recorded Stx-production traits under non-induced culture growth in LB and under phage mobilizing conditions by adding sublethal doses of MMC to the standard LB medium (Figure 8). In all cultures, toxin production was significantly elevated when grown in phage-inducing LB + MMC media. LB titers were undistinguishable between the cultures. In contrast, we observed culture-level differences in Stx-production capabilities upon MMC treatment. More specifically, we noted a correlation of Stx-levels to the respective *stx* status patterns of the strains. The class of ΦStx_2a_ phages carry a highly potent cytotoxin (Fuller et al., 2011; Hauser et al., 2020; Pinto et al., 2021) and are known to mobilize upon activation of the SOS-response (Bonanno et al., 2016; Zhang et al., 2019; Eppinger et al., 2022). In consequence, the Stx titers of the three *stx_2a_* + isolates were all found exacerbated (Figure 8). Strain BAA-2219 (O121:H19), carrying only *stx_2a_*, is the highest-level producer followed by *stx_1a_*/*stx_2a_ +* strains BAA-2440 (O111:H8) and BAA-2196 (O26:H11). Significantly lower and comparable titers were found in the remainder of strains: *stx_1_* strains BAA-2215 (O103:H11) and BAA-2193 (O45:H2), as well as *stx_1a/_stx_2d_* strain BAA-2192 (O145:NM). One caveat using this methodology is that it cannot distinguish between the contribution of individual ΦStx-phages to the overall Stx titer (Skinner et al., 2014). For this reason, we further investigated the mobilization of individual ΦStx-phages and resulting *stx* expression in the three strains that co-harbor ΦStx_1a_, ΦStx_2a_, and ΦStx_2d_ phages (Supplementary Figure S4). Both phages carried by *stx_1a/_stx_2a_ +* strains BAA-2196 (O26:H11) and BAA-2440 (O111:H8) respond to MMC treatment (Supplementary Figure S4). In the latter, ΦStx_2a_ copies and *stx_2a_* transcripts exceed the respective ΦStx_1a_ numbers in both media, while in strain BAA-2196 the *stx_1a_* and *stx_2a_* transcript copies are comparable under non-induced growth in LB. In contrast, only the ΦStx_1a_ phage is significantly mobilized in *stx_1a/_stx_2d_ +* strain BAA-2192 (O145:NM), and in consequence *stx*_1a_ transcripts surpass *stx*_2d_ copies upon MMC induction, while *stx*_2d_ copies are more abundant under non-induced growth in LB. Our observations suggest a considerable heterogeneity in ΦStx-phage mobilization, even within the same ΦStx-phage subtype (Muniesa et al., 2004; Yano et al., 2023). Overall, we observed a positive correlation between phage mobilization, toxin transcript levels, and produced titers (Figure 8; Supplementary Figure S4); in analogy to other studies (de Sablet et al., 2008; Bielaszewska et al., 2012).

## 4 Discussion and conclusions

Non-O157 STEC are a heterogenous group of isolates. The clinically most relevant serogroups, O26, O103, O111, O45, O121, and O145, are colloquially referred to as the “Big Six” due to the rising incidence of human infections. Integration of genome and virulence information for these emerging lineages is critical to improve risk assessment, biosurveillance, and prevention strategies (Franz et al., 2014; Eppinger and Cebula, 2015; Sadiq et al., 2015; Rusconi and Eppinger, 2016). Our study of these ATCC reference type cultures, comprised of six strains representing each of the non-O157 Big Six serogroups, can only provide a glimpse into the genome composition and virulence features. Our future efforts are directed to profile larger strain sets, anchored by the here presented genomes, in an attempt to capture the extent of plasticity found in the emerging human pathogenic Big Six serogroups. Comprehensive analyses of this panel highlight the distinct ΦStx-phage subtypes and their dissimilar phage mobilization patterns, likely associated with the plasticity of regulator regions relevant for replication (Ogura et al., 2015; Llarena et al., 2021; Allué-Guardia et al., 2022; Fagerlund et al., 2022), and intimately linked to Stx-production and Stx-conferred virulence. The different plasmid types and gene contents, including colicin types E3 and D and several antibiotics resistance determinants, provide only a glimpse into the genomic plasticity that can be found in this heterogenous panel of non-O157 STECs (Cortimiglia et al., 2020). Production of colicins and antibiotic resistance are major drivers of microbial evolution (Feldgarden and Riley, 1999; Leekitcharoenphon et al., 2021). Fitness effects mediated by colicins and antibiotic resistance determinants will impact a strain’s individual evolutionary trajectory, and we note that antibiotic resistance and thus pathogenic potential among all STEC serogroups has increased over time and calls for enhanced biosurveillance (Mukherjee et al., 2021). The availability of closed high-quality genomes and carried plasmids of representative Big Six strains, along with insight into their pathogenome make-up and Stx-virulence pathotypes provides a foundation for the research community to broadly explore common and lineage-specific characteristics and evolutionary trajectories of these globally emerging human pathogenic non-O157 STEC lineages.

## Data availability statement

The datasets presented in this study can be found in online repositories. The names of the repository/repositories and accession number(s) can be found in the article/Supplementary material.

## Author contributions

AK: Writing – review & editing, Data curation, Formal analysis, Investigation, Validation, Visualization. SK: Writing – review & editing, Formal analysis, Investigation, Project administration, Validation. JaB: Writing – review & editing, Formal analysis, Investigation, Resources. JoB: Writing – review & editing, Formal analysis, Investigation, Resources. ME: Writing – original draft, Writing – review & editing, Conceptualization, Data curation, Formal analysis, Funding acquisition, Investigation, Project administration, Resources, Software, Supervision, Visualization.

## Footnotes

^1^ https://www.atcc.org/products/mp-9

^2^ http://www.bioinformatics.babraham.ac.uk/projects/fastqc

^3^ https://github.com/tseemann/ABRicate

^4^ https://cge.cbs.dtu.dk/services/ResFinder/

^5^ https://enterobase.warwick.ac.uk/species/ecoli/download_7_gene
