# Supplementary figures and images for: Pathogenomes and virulence profiles of representative big six non-O157 serogroup Shiga toxin-producing Escherichia coli
Source: Front Microbiol. 2024 Mar 18;15:1364026. doi: 10.3389/fmicb.2024.1364026 (PMC10982417; doi:10.3389/fmicb.2024.1364026)

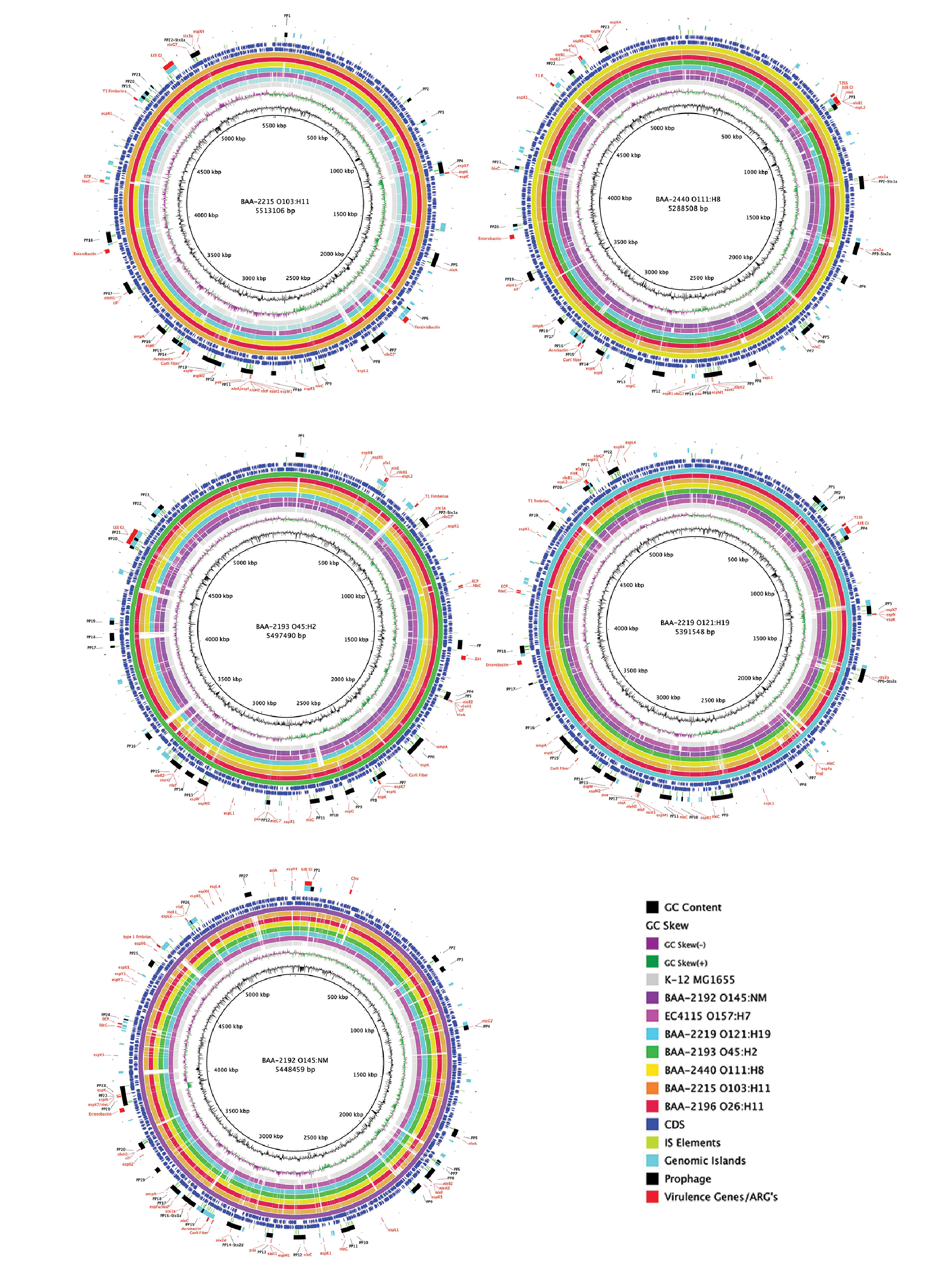

Supplement: Supplementary file 7 [file Image_1.TIF]

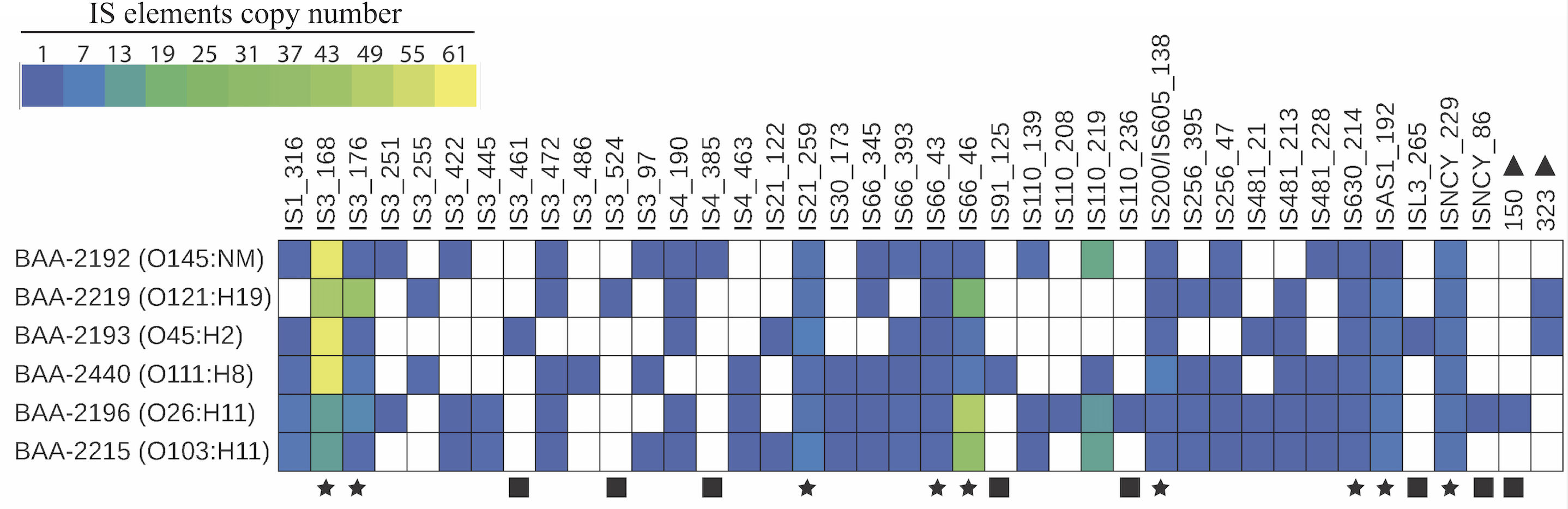

Supplement: Supplementary file 9 [file Image_3.TIF]

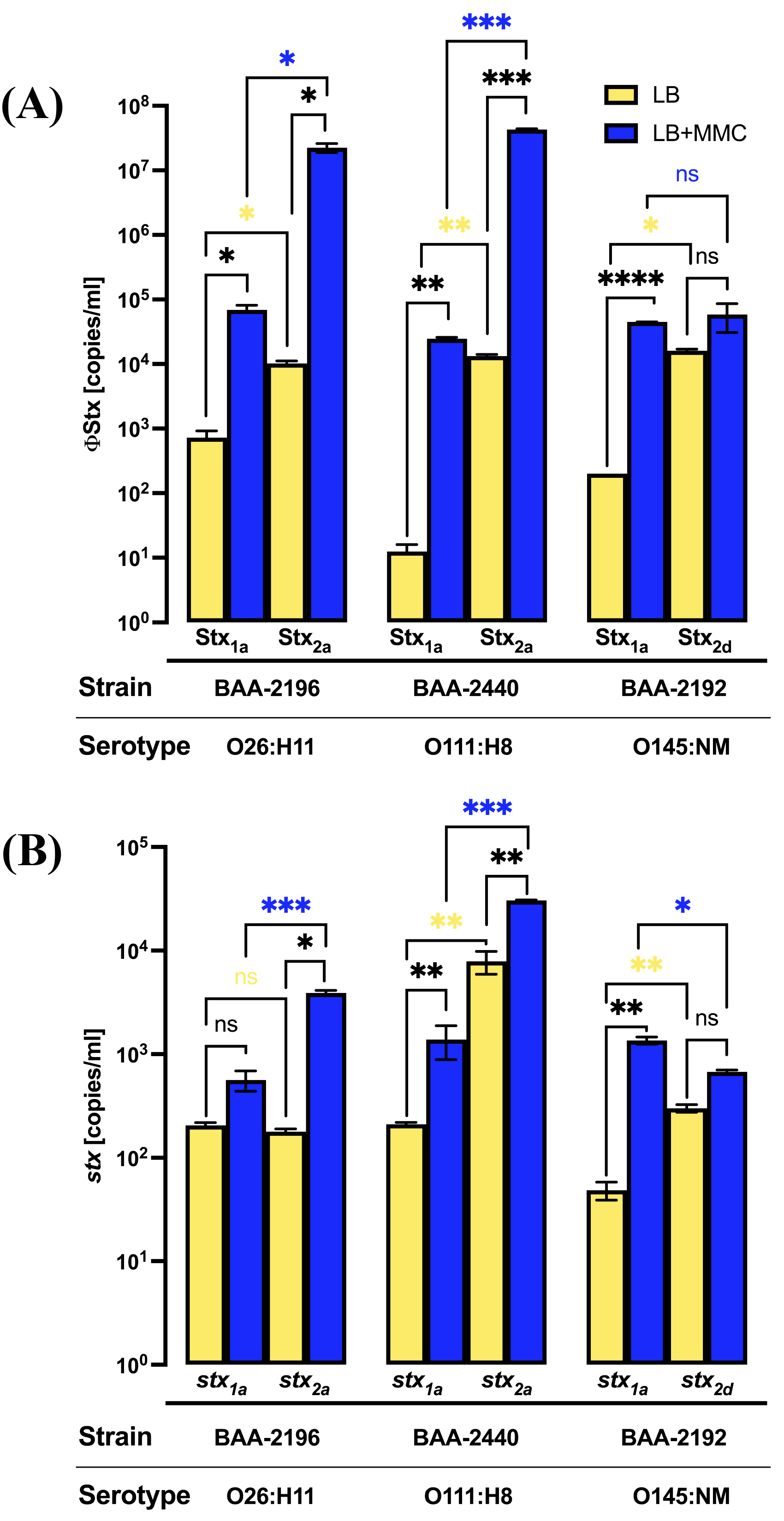

Supplement: Supplementary file 10 [file Image_4.TIF]
